# Supplementary material for: Evaluation of the Antimicrobial Activity of an Extract of Lactobacillus casei-Infected Hermetia illucens Larvae Produced Using an Automatic Injection System
Source: Animals (Basel). 2020 Nov 16;10(11):2121. doi: 10.3390/ani10112121 (PMC7696172; doi:10.3390/ani10112121)
Supplement: Supplementary file 1 [file animals-10-02121-s001.pdf]

Supplementary Materials

Evaluation of the Antimicrobial Activity of an Extract of *Lactobacillus casei*-infected *Hermetia illucens* Larvae Produced using an Automatic Injection System

Kyu-Shik Lee, Eun-Young Yun and Tae-Won Goo

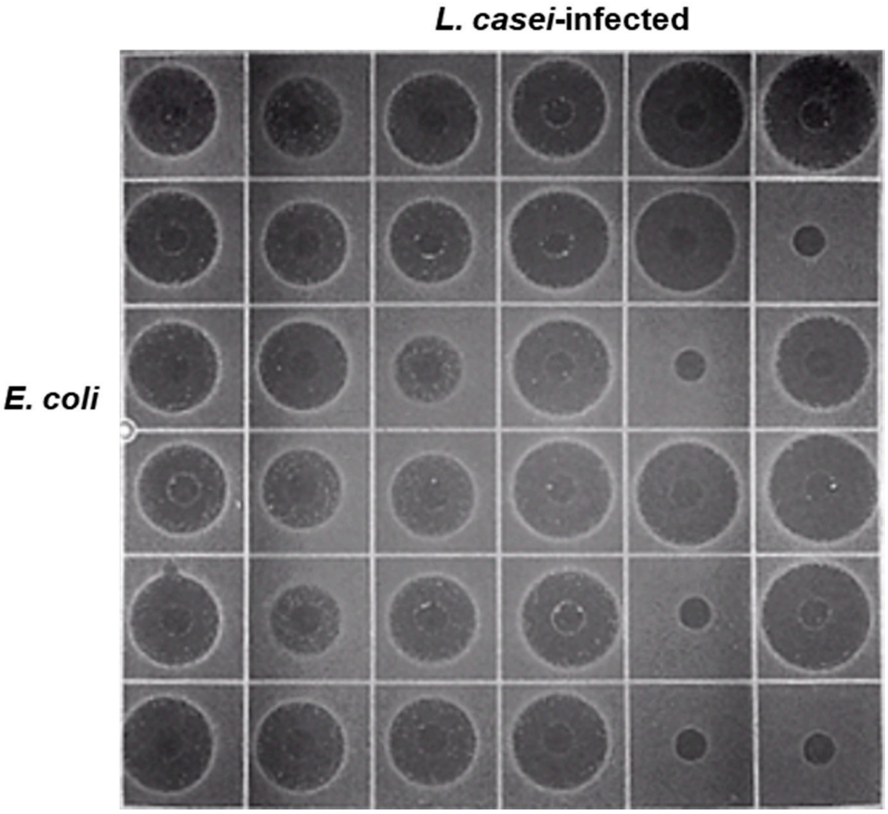

Figure S1. RDA results of the antimicrobial activities of iHIL-E against *E. coli*. *L.casei* were injected into HIL by manual method and held at room temperature for 24 h.

© 2020 by the authors. Licensee MDPI, Basel, Switzerland. This article is an open access article distributed under the terms and conditions of the Creative Commons Attribution (CC BY) license (http://creativecommons.org/licenses/by/4.0/).
